# Longitudinal cohort study of depression, post-traumatic stress, and alcohol use in South African women who attend alcohol serving venues

**DOI:** 10.1186/s12888-014-0224-9

**Published:** 2014-08-06

**Authors:** Laurie A Abler, Kathleen J Sikkema, Melissa H Watt, Lisa A Eaton, Karmel W Choi, Seth C Kalichman, Donald Skinner, Desiree Pieterse

**Affiliations:** Duke Global Health Institute, Duke University, Duke Box 90519, 27708 Durham, NC USA; Department of Psychology and Neuroscience, Duke University, Durham, NC USA; Department of Human Development and Family Studies, University of Connecticut, Storrs, CT USA; Department of Psychology, University of Connecticut, Storrs, CT USA; Unit for Research on Health and Society, Stellenbosch University, Tygerberg, South Africa

**Keywords:** Alcohol use, Depressive symptoms, PTSD symptoms, South Africa, Women

## Abstract

**Background:**

In South Africa, alcohol use poses a public health burden. Hazardous alcohol use often co-occurs with psychological distress (e.g., depression and post-traumatic stress). However, the majority of the research establishing the relationship between alcohol use and psychological distress has been cross-sectional, so the nature of co-occurring changes in psychological distress and alcohol use over time is not well characterized. The objective of this study is to examine the longitudinal relationship between psychological distress and alcohol use among South African women who attend alcohol serving venues.

**Methods:**

Four waves of data were collected over the course of a year from 560 women in a Cape Town township who attended drinking venues. At each assessment wave, participants reported depressive symptoms, post-traumatic stress symptoms, and alcohol use. Multilevel growth models were used to: 1) assess the patterns of alcohol use; 2) examine how depressive symptoms uniquely, post-traumatic stress symptoms uniquely, and depressive and post-traumatic stress symptoms together were associated with alcohol use; and 3) characterize the within person and between person associations of depressive symptoms and post-traumatic stress symptoms with alcohol use.

**Results:**

Women reported high levels of alcohol use throughout the study period, which declined slightly over time. Post-traumatic stress symptoms were highly correlated with depressive symptoms. Modeled separately, both within person and between person depressive and post-traumatic stress symptoms were uniquely associated with alcohol use. When modeled together, significant between person effects indicated that women who typically have more post-traumatic stress symptoms, when controlling for depressive symptoms, are at risk for increased alcohol use; however, women with more depressive symptoms, controlling for post-traumatic stress symptoms, do not have differential risk for alcohol use. Significant within person effects indicated an interaction between depressive and post-traumatic stress symptoms; women reported more alcohol use than usual at times when they had higher post-traumatic stress symptoms, and this increase in alcohol use was further exacerbated for women who also had higher depressive symptoms than usual.

**Conclusions:**

These findings suggest that interventions targeting post-traumatic stress, especially when post-traumatic stress is comorbid with depression, may reduce alcohol use among South African women who drink.

## Background

Globally, alcohol use is a mental health problem that raises significant public health concern, ranking third in its contribution to the global burden of disease [1]. Problematic alcohol use is linked to numerous disease and behavioral outcomes (e.g., fetal alcohol spectrum disorder, gender-based violence, and HIV risk behavior) [2-4], especially among women [5]. To develop effective alcohol interventions for women, further understanding is needed regarding the factors associated with problematic alcohol use, specifically in high-risk settings such as South Africa.

South Africans who drink have some of the highest levels of alcohol use in the world, with annual consumption rates estimated at 16.6 liters per drinker, and a high prevalence of women (23.8%) who use alcohol [6], often at hazardous levels [7]. The high levels of alcohol use can be contextualized within South Africa’s racial and social history. As a result of historic race-based policies, illegal drinking establishments, called *shebeens*, emerged as popular places in non-white communities where patrons gathered to socialize and drink [8]. Additionally, the *dop* system to pay non-white farm workers with alcohol in lieu of monetary wages left behind a legacy of problematic alcohol use and dependence, particularly among women [9]. Women from the Western Cape, a wine growing region where *dop* payments were widely used, report some of the highest levels of alcohol use compared to other parts of South Africa [10].

Many South African women live in impoverished settings where life is fraught with experiences of violence, unemployment, and gender inequality [11,12], which may increase vulnerability to psychological distress, such as depression and post-traumatic stress disorder (PTSD). Previous research has shown that a significant portion of the South African population have reported depressive disorder or PTSD [13]. These reports are likely higher among people who use alcohol, people living in impoverished townships outside of urban cities, and women [14,15]. Psychological distress in South Africa often goes undetected, and even when individuals receive a diagnosis, the public health care system is not fully equipped to provide comprehensive treatment [16]. Without treatment, the distress symptoms are dynamic and long-lasting, with one study finding that PTSD symptoms resulting from trauma persist, on average, for nearly five years [15]. Not only do the symptoms of depression and PTSD persist, but depression is highly likely to co-occur with PTSD [17]. Comorbid depression and PTSD are especially common among women who have experienced multiple traumas [18] or gender-based violence [19]. Multiple forms of psychological distress compound each other, such that women with comorbid depression and PTSD suffer from much greater impairment relative to women with depression alone [20].

The presence of persistent psychological distress can exacerbate alcohol use [21]. South African women who drink may do so to cope with psychological distress that results from problems influenced by the social context – such as traumas [22], poverty [23], and gender-based violence [24]. A high co-occurrence of psychological distress and alcohol use has been shown in Western settings [25]. Longitudinal studies have found that depression and alcohol use co-occur over time [26], and that increased depression leads to greater alcohol use [27]. Similarly, longitudinal studies have shown that PTSD and alcohol use are reciprocally related over time [28] and that PTSD can lead to greater alcohol use [29]. In South African settings, evidence for these relationships is also emerging [30]. Psychological distress and alcohol use has been suggested theoretically [31] and empirically [26] as being bidirectional and reciprocal. The strongest evidence of causal relationships from longitudinal studies has found that alcohol use increases the incidence and severity of psychological distress [32]. Longitudinal studies have also found that when problematic drinking is used to cope with psychological distress, there are worse long-term outcomes for mental health and substance use [31].

While drinking may be used to cope with life stressors [33], little is known about how psychological distress influences the patterns of alcohol use over time among South African women who use alcohol. In particular, few studies have investigated: 1) the differential effect of psychological distress (depression only, PTSD only, or both) on alcohol use; and 2) how an individual’s change in psychological distress relates to her alcohol use (i.e., within person effects). For the first gap, while depression and PTSD have both been shown to be associated with problematic alcohol use [25], less is known about how these two types of psychological distress work separately and in tandem to drive the use of alcohol, especially among South African women who drink. For the second gap, much of the research that explores drinking as a means to cope with distress comes from cross-sectional studies which can only elucidate between person effects, i.e. the mean differences in alcohol use that occur between individuals. Meanwhile, qualitative research suggests that South African women drink more than usual after experiencing stressors [33]. However, this has not been examined quantitatively in this setting and less is known about the within person effects between psychological distress and alcohol use. Longitudinal studies are necessary to characterize the dynamic within person drinking effects associated with episodes of psychological distress.

This study addresses these research gaps by exploring how depressive and PTSD symptoms were associated with patterns of alcohol use over time. Using longitudinal data from a cohort of women who attend drinking venues in a township of Cape Town, South Africa, the current study is guided by two objectives. The first objective is to examine both the unique and relative contributions of depression and PTSD on alcohol use patterns. The second objective is to test whether heightened psychological distress within an individual is associated with heightened alcohol use (within person effects) and whether participants who have higher psychological distress in general have higher levels of drinking (between person effects).

## Methods

### Procedures and sample

This longitudinal study was based in Delft, a peri-urban township located about 15 miles east of Cape Town, South Africa in the Western Cape Province. Delft was established over twenty years ago. It is inhabited by an approximately equal number of individuals who are Xhosa-speaking Black Africans and individuals who are Afrikaans-speaking Coloureds, an accepted racial category in South Africa that denotes historic mixed ancestry. To locate alcohol serving venues in Delft, study staff used an adaptation of the Priorities for Local AIDS Control Efforts method (PLACE) [34] to conduct street intercept interviews. These interviews identified 124 venues that were assessed by study staff for eligibility (seating for patrons, >50 unique visitors per week, >10% female patrons, and willingness to have the research team regularly visit). The final twelve study venues were selected to represent an equal number of venues with predominantly Black or Coloured patrons, a geographic range throughout the township, and a mix of large taverns (licensed) and small *shebeens* (unlicensed).

Female patrons from the study venues were recruited into a multi-wave prospective cohort. Participants were eligible if they were at least 18 years old (the legal drinking age in South Africa), regular patrons at one of the study venues, currently lived in Delft, and planned to live in Delft for the next year. Participants were enrolled from June 2009 - May 2010.

Four assessment waves were collected (baseline, 4-, 8-, and 12-months). At each assessment, trained data collectors administered an Audio Computer-assisted Self-interviewing (ACASI) survey to the participants. Prior to the administration of the first assessment, all participants provided informed consent. All data collection procedures were approved by the Institutional Review Boards at the University of Connecticut and Duke University, and the Human Research Ethics Committee at Stellenbosch University.

A sample of approximately 50 women per venue was recruited from each of the 12 study venues (actual range across venues: 26–65 participants). Study staff invited 604 women in the alcohol-serving venues to participate. Of those, 560 women (92.7%) completed the baseline assessment. Participants were retained at high rates throughout the study (96.3%, 95.0%, and 94.6%, respectively, for each remaining assessment) through rigorous tracking and communication procedures. Participants lost to follow up moved out of Delft or could no longer be located. Those lost to follow up were more likely to be older, but did not differ by depressive symptoms, PTSD symptoms, alcohol use and race compared to participants who were retained.

### Measures

Measures included demographic characteristics, psychological distress (depressive and PTSD symptoms), and alcohol use. The demographic characteristics were measured at the first assessment. The measures of alcohol use, depressive symptoms, and PTSD symptoms were time-variant and collected at all four waves. Throughout the paper, references to the depression and PTSD measures indicate the degree of symptoms, rather than a clinical diagnosis.

### Demographic characteristics

Participants reported their race, age, education, employment, relationship status, and number of children.

*Depressive symptoms* were assessed using the Center for Epidemiological Studies Depression Scale (CES-D) [35], which is comprised of 20 items regarding the frequency of depressive symptoms in the past week. Response categories ranged from zero (never/rarely) to three (most of the time/all of the time), possible range 0–60. Cronbach’s α coefficients ranged from 0.90 to 0.91 over the four assessment waves. The intraclass correlation (ICC) for depression was 0.66, representing both the proportion of variance in depression between persons out of the total variance, as well as the correlation within the individual’s depression scores across the four assessments. For the purposes of descriptive analysis, a score of ≥16 on the CES-D indicated significant depressive symptoms [36].

*PTSD symptoms* were measured with the PTSD Checklist-Civilian Version (PCL-C) [37]. Participants responded to 17 items that assessed the frequency of experiencing the DSM-IV symptoms of PTSD in the past month. Response categories ranged from one (not at all) to five (very often). The summary score ranges from 17–85, with higher scores indicating greater severity. The internal consistency reliability (Cronbach’s α) ranged from 0.95-0.97. The ICC for stress symptoms was 0.60. For descriptive analysis, a score of ≥50 on the PCL-C indicated significant PTSD symptoms [37].

*Alcohol use* was the study outcome assessed by the Alcohol Use Disorders Identification Test, version C (AUDIT-C) [38]. The AUDIT-C is comprised of three questions that ask about current drinking frequency (“how often do you have a drink containing alcohol”; 0 = ‘never’ to 4 = ‘more than four times a week’); number of drinks per occasion (“how many drinks containing alcohol do you have on a typical day when you are drinking”; 0 = ‘1 or 2’ to 4 = ‘10 or more’), and frequency of binge drinking occasions (“how often do you drink six or more drinks on one occasion”; 0 = ‘never’ to 4 = ‘daily or almost daily’). Items were summed to create the alcohol use score (possible range 0–12), with higher scores indicating more problematic alcohol use. Participants who reported that they abstained from drinking were given a score of zero. Cronbach’s α coefficients ranged from 0.73-0.80. The ICC for alcohol use was 0.59. For the descriptive analysis, as long as all the points did not result from the drinking frequency question, a score of ≥3 for women on the AUDIT-C is typically used as a cut-point to indicate hazardous drinking [39].

### Analysis

The longitudinal effects of psychological distress on alcohol use patterns were estimated with multilevel growth models [40]. The models were specified at two levels in which time (level one) was nested within individuals (level two). This modeling separated the total variance in the outcome into within person (the level one model, i.e., an individual’s variation in alcohol use over time) and between person variation (the level two model, i.e., mean differences in alcohol use). To model the variance components, participants were treated as random effects, allowing for variation in individuals’ initial level of alcohol use (the intercept) and linear change in alcohol use over time (the slope). All of the other variables in the models (e.g. mental health, demographic covariates) were treated as fixed effects.

Prior to conducting the longitudinal analyses, the independent variables were centered to evaluate the individuals’ mean trajectories of alcohol use and individuals’ deviations from their personal mean trajectories. As a level one variable, time acted as the metric of change and was centered so that the intercept represents the average level of the alcohol use outcome at the first assessment. The time-varying predictors, depression and PTSD, were separated into two variables to represent within person (level one) and between person (level two) variation. At level one, the time-varying depression and PTSD variables were person-mean centered by subtracting the mean for the individual from her score at each assessment. At level two, the person-mean values were calculated by averaging an individual’s predictor scores and subtracting this value from the sample mean. The level two age covariate was grand-mean centered relative to the participants’ ages reported at the first assessment. The level two race covariate compared Black participants to Coloured/Other participants as the reference group.

Multilevel growth models were calculated using PROC MIXED (SAS, v9.3, Cary, NC) to estimate the alcohol use patterns in a series of three steps. In each of the models, full information maximum likelihood was used to handle the missing data resulting from loss to follow-up. *Step one:* unconditional growth models evaluated the shape (linear and quadratic) of change over time and the covariance structure of the unconditional trajectories of the alcohol use. Restricted maximum likelihood (REML) was used to distinguish nested models with varying random effects, and maximum likelihood (ML) estimation was used for nested models with varying fixed effects [41]. *Step two*: Within this step, three different models were evaluated to address the first aim of the study: 1) the depression predictor alone; 2) the PTSD predictor alone; and 3) depression and PTSD predictors together. For each of these three models, the main effects were tested for the within and the between person psychological distress variable(s) on alcohol use. Each of the three models was adjusted for race and age, which have been shown to be associated with alcohol use among South Africans [7]. The likelihood ratio test (LRT) was used to determine model fit when the models were nested in parameters. *Step three:* for each of the three models (depression alone, PTSD alone, and depression and PTSD together), an interaction model was developed to address the second aim of the study. Potential two-way and three-way interactions were tested among the time variable, the covariates, and the within and between person mental health variables. The criterion for retaining interaction terms in the model was based on their significance at the level of p < 0.05 and only significant interactions are reported in the results.

## Results

### Characteristics of the sample

The participants were primarily Coloured (n = 343, 61%), followed by Black (n = 204, 36%), White (n = 11, 2%) and Indian (n = 2, <1%). On average, the women were 33.9 (SD = 11.6) years old. Most participants had not finished high school (n = 457, 82%) and were unemployed (n = 414, 74%). About a third of the women were married or living with their partner (n = 199, 36%) and most had at least one child (n = 452, 81%). Five of the women reported abstaining from drinking for the entire duration of the study.

### Descriptive statistics

Table 1 presents the means, standard deviations, and prevalence (according to the established cut-offs) for depression, PTSD, and alcohol use at each wave. Across waves, 69-71% met the criteria for depressive disorder (CES-D ≥16), 17-21% met the criteria for PTSD (PCL-C ≥50), and 75-86% met the criteria for hazardous drinking (AUDIT-C ≥3). At each wave, between 28-31% reported neither significant depressive nor significant PTSD symptoms, and 51-54% reported having significant depressive but not significant PTSD symptoms. PTSD nearly always co-occurred with depression; at each time point, less than 1% reported significant PTSD without significant depressive symptoms. Between 17-21% reported significant PTSD (whether with or without co-occurring significant depressive symptoms). Across waves, the correlations between continuous measures ranged from: 1) 0.66-0.72 (p < 0.001) for depression and PTSD; 2) 0.17-0.22 (p < 0.001) for depression and alcohol use; and 3) 0.17-0.24 (p < 0.001) for PTSD and alcohol use.

**Table 1:** **Descriptive statistics of depressive symptoms, post-traumatic stress symptoms, and alcohol use scores by assessment wave**

	**Wave 1 (Baseline)**		**Wave 2 (4-month)**		**Wave 3 (8-month)**		**Wave 4 (12-month)**	
	**n = 560**		**n = 538**		**n = 530**		**n = 527**	
**Variable**	**M (SD)**	**%**	**M (SD)**	**%**	**M (SD)**	**%**	**M (SD)**	**%**
Depression	23.2 (12.2)	71.3	23.1 (12.0)	70.3	22.9 (12.0)	68.9	22.6 (11.7)	69.5
PTSD	36.3 (16.3)	20.9	35.5 (16.1)	18.8	35.2 (15.1)	17.4	34.1 (15.9)	17.3
Alcohol use	5.7 (2.9)	85.6	5.1 (2.9)	80.6	4.9 (3.0)	75.9	4.7 (3.0)	75.1

Mean scores were calculated prior to centering the variables. Percentages were calculated as the proportion of the participants reporting significant depressive symptoms (CESD ≥ 16), PTSD symptoms (PCL-C ≥ 50), or hazardous drinking (AUDIT-C ≥ 3) at each assessment.

### Patterns of alcohol use (unconditional model)

The best fitting unconditional model for alcohol use included a fixed quadratic time effect, and a random linear intercept and slope with heteroscedastic residual error variance (Table 2). As shown by the random effects, there was significant variance between women in mean levels of alcohol use at Wave 1 (intercept estimate = 4.74, p < 0.001, 95% CI = 1.358, 9.895). There was also significant variance between women in the alcohol use slope over time (linear slope estimate = 0.24, p < 0.001, 95% CI = −1.579, 0.320). The coefficients for the fixed effects for the time (linear) and the time x time (quadratic) variables, indicates that there were significant linear decreases (time estimate *= −*0.63, SE = 0.12, p < 0.001) in the predicted mean trajectory of alcohol use which decelerated (time *x* time estimate = 0.11, SE = 0.04, p < 0.001) over the four waves.

**Table 2:** **Unconditional growth model for the patterns of alcohol use**

**Parameter**	**Unconditional growth model**
*Random effects*	
Intercept	4.743*** (0.364)
Linear slope	0.235*** (0.053)
*Fixed effects*	
Intercept	5.626*** (0.120)
Linear time	−0.630*** (0.123)
Time x Time	0.112** (0.038)

Residual errors were allowed to vary over time and were significant (p <0.001) across all assessments. **p < 0.01; ***p < 0.001.

### Depression (only) associated with alcohol use

The within person and between person depression variables, controlling for race and age, were added as fixed effects to the unconditional model (Table 3 – depression only model). No significant moderation was found for potential interaction terms, so only the model from Step two is reported. This model fit the data significantly better than the unconditional model (LRT difference: *Χ*^2^ = 32, df = 3, p <0.001). The intercept predicts that the alcohol use score would be 4.504 (SE = 0.281, p < 0.001) for a woman at the first assessment, who had the mean level of depression in the sample and was experiencing her individual-average level of depression. For within person depression, during a time point when a woman reported a unit increase from her typical (i.e. individual-average) depression score, her alcohol use score would increase by 0.02 (SE = 0.007, p < 0.01), suggesting a difference of 0.2 in the AUDIT-C score for woman reporting a 10-point difference in her CES-D score between waves. For between person depression, for every one unit higher that a woman reported for her typical depression score, her alcohol use score would be 0.06 units (SE = 0.01, p < 0.001) higher than a woman who scored one unit lower.

**Table 3:** **Results for the multilevel growth models of the patterns of alcohol use**

**Parameter**	**Depression only model**	**PTSD only model**	**Depression + PTSD model**
*Time components*			
Intercept	4.504*** (0.281)	4.880*** (0.199)	4.772*** (0.283)
Time	−0.627*** (0.123)	−0.620*** (0.123)	−0.616*** (0.123)
Time x Time	0.113** (0.038)	0.113** (0.038)	0.111** (0.038)
*Psychological distress*			
WP depression	0.020** (0.007)		0.012 (0.007)
BP depression	0.060*** (0.010)		0.007 (0.015)
WP PTSD		0.014** (0.005)	0.009ǂ (0.005)
BP PTSD		0.056*** (0.007)	0.051*** (0.012)
WP depression x WP PTSD			0.002** (0.001)
Pseudo-R^2^	0.086	0.107	0.112

Notes: Within person (WP) depression and WP PTSD were time-varying and person-mean centered. Between person (BP) depression and BP PTSD used the person mean scores. All models controlled for age (grand-mean centered) and race (reference = Coloured/Other). ǂp < 0.10; **p < 0.01; ***p < 0.001.

### PTSD (only) associated with alcohol use

The within person and between person PTSD variables, controlling for race and age, were added as fixed effects to predict alcohol use trajectories (Table 3 – PTSD only model). No significant interactions were found between any of the variables in the model, so only the model from Step 2 is reported. This model fits the data significantly better than the unconditional growth model (LRT difference: *Χ*^2^ = 32.2, df = 3, p <0.001). For women at the first assessment who had the mean level of PTSD in the sample and were experiencing their individual-average level of PTSD, the intercept predicts that their alcohol use score would be 4.880 (SE = 0.199, p < 0.001). For within person PTSD, during a time point when a woman reported a unit increase from her individual-average PTSD score, her alcohol use score would increase by 0.01 (SE = 0.005, p < 0.01). For between person PTSD, for every one unit higher that a woman reported for her typical PTSD score, her alcohol use score would be 0.06 units (SE = 0.01, p < 0.001) higher than a woman who scored one unit lower.

### Depression and PTSD associated with alcohol use

Within person and between person depression and PTSD, controlling for race and age, were entered together into the model predicting alcohol use trajectories (Table 3 — depression and PTSD model). For women who were experiencing their usual amount of depression and PTSD, and reported the mean level of depression and PTSD scores in the sample, the model predicted an alcohol use score of 4.780 (SE = 0.284, p < 0.001). At the between person level, increases in PTSD (b = 0.051, SE = 0.012, p < 0.001), but not depression (b = 0.007, SE = 0.015, p = n.s.), were associated with greater risk for more alcohol use. That is, when both between person PTSD and depression were included in the model, the effects of between person PTSD drove the risk of increased alcohol use and between person depression was no longer significant in the model.

**Figure 1:**
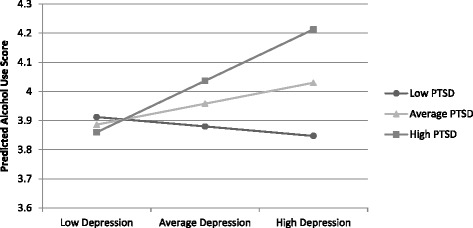
**Estimated alcohol use as a function of within-person depression and PTSD interaction at low (−1 standard-deviation), average, and high (+1 standard deviation) levels of the interaction.**

This final model included an interaction term for within person depression by within person PTSD, but no other interactions (among time, race, age, and within person and between person depression and PTSD) were significant. This association between the within person depression by within person PTSD interaction and alcohol use was small, but significant (b = 0.002, SE = 0.001, p < 0.01). The positive relationship for within person PTSD (i.e., waves when women report heightened PTSD) and alcohol use was more pronounced among women who were also experiencing heightened (within person) depression. The interaction for within person depression and within person PTSD was probed in Figure 1 to visualize these relationships. Predicted alcohol use scores were graphed for low (−1 standard deviation), average, and high (+1 standard deviation) within person depression and PTSD scores.

## Discussion

Using longitudinal data, this study explored the association between psychological distress and alcohol use over time in a cohort of South African women attending drinking venues. Not surprisingly, we found that women who attended these venues had high levels of hazardous alcohol use and depression. PTSD was less prevalent, but still prominent, and nearly all participants with PTSD reported comorbid depression. Our longitudinal data revealed that individual women’s problematic alcohol use was higher at times when they experienced increases in psychological distress.

Over the study period, mean alcohol use decreased over time in a curvilinear fashion. When depression and PTSD were included in the longitudinal model separately, greater average levels of the psychological distress and heightened episodes of distress were associated with more alcohol use. When depression and PTSD were included together in the longitudinal model, the mean levels of PTSD, but not depression (between person effects), were significantly associated with greater alcohol use. Mean differences in PTSD between individuals, and likely the high correlation of PTSD with depression, seemed to drive the overall levels of alcohol use. The within person variables, examining episodes of heightened depression and PTSD, showed a slight, but significant interaction effect on alcohol use. This finding suggests that women are engaging in episodes of increased alcohol use at times when they are also experiencing more co-occurring depression and PTSD than usual.

The reported levels of alcohol use were high, but not unexpected given research showing that venue attendance is normative for social engagement and that women who frequent venues often exhibit problematic drinking [22]. Our findings demonstrated that alcohol use decreased at a rate that slowed over time. These decreases, though statistically significant, approximated a one unit reduction in the alcohol use score over the course of the study. Thus, despite this reduction over time, most women reported drinking throughout the study at hazardous levels. Although these data cannot identify why alcohol use declined in the curvilinear (quadratic) fashion, potential explanations include regression to the mean in a sample of predominantly hazardous drinkers, testing effects from the multiple assessments (on psychological distress symptoms, alcohol use or both), and maturation of the women over time.

Findings revealed that psychological distress was pronounced among our sample and comparable to nationally representative samples assessing depression and PTSD as disorders [13], especially among alcohol users in South Africa [30,42]. Depression was more common than PTSD. Among the women who had significant PTSD symptoms, nearly all reported comorbid significant depressive symptoms. However, many of the women with significant depressive symptoms did not report significant PTSD symptoms. The high likelihood that PTSD co-occurs with depression is similar to findings from other studies with vulnerable women [43].

When depression and PTSD were modeled for their unique (i.e., not adjusted for the other distress) association with alcohol use, two findings emerged. First, episodes of heightened distress (within person) were associated with episodes of increased alcohol use, and second, higher levels of overall distress (between person) were associated with greater overall alcohol use. When both depression and PTSD were included in the model, participants with higher PTSD, but not higher depression, used significantly more alcohol. Therefore, the significant effects of depression on alcohol use in the depression-only model, likely resulted from depression’s co-occurrence with PTSD. The strong correlation between PTSD and depression scores partially hinders the interpretation of the contribution of PTSD on alcohol use that is separate from depression. However, some insight about the joint effects of PTSD and depression can be found from the significant interaction – for episodes of PTSD by episodes of depression (within persons) – which demonstrate that the greatest alcohol use happens during co-occurring episodes of heightened PTSD and depression. Further, because most women in the sample reported hazardous drinking and significant psychological distress symptoms throughout the study period, the reductions seen in psychological distress were not large enough to be associated with clinically meaningful decreases in alcohol use below hazardous levels, i.e., the decreases in alcohol use associated with reduced psychological distress were modest. Nevertheless, the findings suggest that clinically meaningful reductions in the psychological distress and alcohol use could result from concerted intervention to reduce their co-occurrence.

This study provides directions for future research. One avenue is to explore the impact of diverse stressors in this setting, including trauma and abuse, on the mean levels and episodes of distress. Previous research in South Africa has shown that multiple stressors compound each other, leading to more psychological distress symptoms [18], which in turn increase drinking [22]. Research is needed to evaluate how the characteristics and synergy of multiple stressors influence the patterns of depression, PTSD, and their comorbidity over time, which then affect alcohol use. Additionally, further research is needed to understand other factors that impact patterns of alcohol use in this setting, given that depression and PTSD accounted for approximately 10% of the variance (denoted by the Pseudo R^2^) in alcohol use. Although drinkers tend to have the most problematic alcohol use outcomes when they drink to cope with psychological distress, many women may also be motivated to drink for social reasons [44]. Because this research was conducted with women attending drinking venues – a social place that can both exacerbate and protect against risk behaviors [45] – there may be other factors related to the social context that motivate women’s alcohol use, which interact with psychological distress.

These findings should be considered in light of potential limitations. All the key variables ascertained sensitive constructs that were captured through self-report. Thus, depression, PTSD, and alcohol use are subject to the limitations of response bias, such as underestimation due to social desirability. Nevertheless, the CES-D, PCL-C, and AUDIT-C are psychometrically valid assessment tools that have been used in South Africa [46-48]. The alcohol use measure asks three questions to ascertain levels of current drinking and may not capture the entirety of drinking consumption over the four-month period between assessments. Participants reported depression in the past week and PTSD in the past month. As a result of these different timeframes, the study can only depict the longitudinal association between psychological distress and the alcohol use to show they significantly co-occur at each wave. Alcohol use could also have impacted psychological distress. Future longitudinal research should consider following women using more comprehensive measures that capture the entirety of the time frame between assessments and more assessment waves to evaluate the impact of psychological distress at one time point on subsequent alcohol use. In addition, other unmeasured factors, such as personality characteristics or new traumatic experiences, could be driving the joint and bi-directional changes in distress and alcohol use. Finally, the sample was non-random, so caution is warranted about generalizing findings to all South African women who drink, especially women who do not attend venues, such as older women who tend to be less likely to attend venues than younger women [49].

Despite these limitations, this study benefits from the high levels of participant retention in exploring the relationships between psychological distress and alcohol use. Although this longitudinal analysis does not establish causal relationships, they can depict temporal changes which provide insight beyond the cross-sectional view. The longitudinal design not only allows for an examination of the between person effects that look at mean level differences, but more importantly, it allows for the examination of within person effects (which cannot be captured in cross-sectional designs) of the dynamic changes in depression and PTSD within an individual woman over time that can contribute to episodes of alcohol use.

The study findings have important implications for policy and intervention for decreasing alcohol use. Reducing drinking has been identified by the Department of Health, together with expanding the screening and treatment of mental health and substance use, as part of their long-term policies for improving the health of South Africans [50]. This study shows high rates of comorbid alcohol use and psychological distress among women who drink, which suggests that a multi-pronged approach is necessary to address alcohol use, psychological distress, and their comorbidity. To reduce alcohol use among women, drinking venues can serve as sites for interventions to identify venue patrons who also have co-occurring psychological distress. To treat psychological distress among individuals who drink in the South African setting, a problem-solving approach has been recommended to build coping skills to address the problems that lead to distress and drinking [51]. In addition, South Africans who experience psychological distress, such as depression and/or PTSD, are in need of greater access to other treatments, such as cognitive behavioral therapy and pharmacotherapy, to treat their distress. Even though simultaneous treatment is not commonly available for many South Africans with co-occurring problematic alcohol use and psychological distress, these findings suggest that integrated treatment [52] could improve both mental health and drinking outcomes. Finally, other interventions are sorely needed that address precursors of drinking and distress, such as traumas and chronic stressors, and therefore decrease the burden of psychological distress and subsequent drinking. Future research to understand these important precursors and how they impact drinking as a coping mechanism may provide further insights into the link between alcohol use and psychological distress among South Africa women.

## Conclusion

In conclusion, this study identifies high levels of psychological distress among women who also drink at hazardous levels. It highlights that PTSD and depression co-occur differentially with drinking over time. Women with overall higher levels of PTSD, which is highly comorbid with depression, are particularly at risk greater alcohol use above hazardous levels. These findings point to the critical need for interventions that jointly address alcohol use and psychological distress among women in South Africa who frequent alcohol-serving venues. Future research should focus on identifying the causal precursors of hazardous drinking and psychological distress for these women that could serve as prevention targets to alleviate both.
